# Electronic innovation and readership

**Published:** 2012-04

**Authors:** AJ Brink

**Affiliations:** The Cardiovascular Journal of Africa (CVJAfrica)

*The Cardiovascular Journal of Africa (CVJAfrica)* has updated its electronic presence with a modern website to provide for quick and easy access to the Journal’s contents and services. Importantly, for narrow-band users in developing regions, the Journal has ensured that access to its articles is direct to text in compact PDFs.

For our advertisers, within both the pharmaceutical and academic environments, the Journal site has links via advertisements to further product details. For authors, their articles are indexed in PubMed, Medline, Embase and Scopus and are tracked by Thompson Reuters (Web of Knowledge – ISI) which provides the Journal’s impact factor.

Reassuringly, in the current debate on ‘open access’, all articles published in the *CVJAfrica* are provided as full text, free of charge on the Pubmed and *CVJAfrica* site. The copyright of published articles is held by the *CVJAfrica* and if these articles are reproduced internationally, payment is made via the US-based Copyright Clearance System. Again, the charge for academic use is extremely low (0.1 American cents per page) while commercial use reflects the actual costs more closely.

Printed versions of the Journal are provided to South African reference libraries and to relevant specialists in South Africa. Increasingly, the Journal is accessed electronically, with 1 800 visitors to the site monthly, who view on average four pages of Journal content. In addition, 2 500 articles are downloaded per month globally by readers using the Pubmed full-text facility.

The challenge for all e-based, independent journals is how to ensure sufficient financial support. We have reluctantly introduced a modest ($50) submission fee to help us cover the significant costs of Editorial Manager; the well-known and highly regarded Aries manuscript submission system. Many open-access journals such as those in BioMedCentral charge a $1 000 author submission fee.

The *CVJAfrica* focus is to publish quality research articles in the field of vascular disease. We thank our authors, reviewers and editors across the country for their support of the Journal’s activities.

